# Single-Session Attention Bias Modification Training in Victims of Work-Related Accidents

**DOI:** 10.3389/fpsyg.2018.01619

**Published:** 2018-09-04

**Authors:** Giulia Buodo, Elisabetta Patron, Simone Messerotti Benvenuti, Daniela Palomba

**Affiliations:** Department of General Psychology, University of Padova, Padova, Italy

**Keywords:** work-related accidents, attentional bias, Attention Bias Modification Training, cognitive dysfunctions, injured workers

## Abstract

Individuals who experienced traumatic work-related accidents frequently show cognitive deficits and biased processing of trauma-relevant information, which, in turn, could increase the risk of further accidents. The attention bias modification training (ABMT) is designed to reduce hypervigilance toward and enhance attentional disengagement from threat stimuli. The aim of the present study was to assess whether it is possible to implicitly reduce the attentional bias toward trauma-related stimuli through a single session of ABMT in individuals who experienced a traumatic occupational accident. Nineteen individuals who had experienced a traumatic work-related accident and 11 workers who never experienced a work accident (control group) underwent a preliminary assessment of cognitive performance (executive functions and sustained attention) and an evaluation of the attentional bias toward accident-related pictures by means of a dot-probe task. The results showed that injured workers performed more poorly than controls in tasks of executive functions and concentration abilities. Also, injured workers showed an attentional bias toward trauma reminders (i.e., faster reaction times to probes replacing trauma-related pictures). Injured workers were then randomly allocated to a single-session of ABMT (*N* = 10) or to an Attention Control Condition (ACC; *N* = 9). After the training, the dot-probe task was administered again to assess changes in the attentional bias toward trauma-relevant pictures. Injured workers who underwent the ABMT, but not those who underwent the ACC, showed a significant reduction of the attentional bias from pre- to post-training. Overall, these results support previous findings reporting an association between traumatic occupational accidents and cognitive dysfunctions. More importantly, these preliminary findings add to a growing body of evidence suggesting the effectiveness of a short ABMT in reducing the attentional bias after a traumatic workplace accident.

## Introduction

European statistics have shown that in 2015 there have been close to 3.1 million non-fatal occupational accidents (Eurostat, 2017). Workers who underwent a traumatic accident often experience depressive, anxiety, and post-traumatic symptoms (Novara et al., 2009; Ghisi et al., 2013; Lin et al., 2014). Moreover, injured workers have been shown to suffer from cognitive impairment in different domains, including memory (Bustamante et al., 2001; Johnsen and Asbjørnsen, 2008) and attention (Buckley et al., 2000; Constans, 2005). Buodo et al. (2011) showed that workers who experienced traumatic accidents have deficits in several cognitive domains, including attention and concentration, memory, perceptual-psychomotor speed, and difficulties in executive functions. Importantly, evidence was found that in these individuals attentional performance is sensitive to emotional interference from trauma-related cues (Buodo et al., 2011). Specifically, a systematic tendency to direct attention toward stimuli that are perceived as dangerous in the external (or internal) environment (increased vigilance toward threatening stimuli), most notably trauma reminders, resulted in biased processing of trauma-related stimuli to the expense of trauma-unrelated cues. According to Foa et al. (1989), the presentation of stimuli associated with the traumatic event activates a specific network encoded in memory, which contains representations of trauma-related stimuli. This cognitive fear structure increases sensitivity and attention toward stimuli recorded in the network and, in turn, the network’s activation results in reduced availability of cognitive resources for the processing of non-trauma-related stimuli. In line with this hypothesis, an attentional bias toward trauma-related information has been shown to predict anxiety and post-traumatic symptoms in individuals exposed to traumatic situations (Wald et al., 2011).

Indeed, two components of the early attentional bias toward threat-related stimuli have been highlighted, specifically, facilitated attentional engagement and impaired disengagement. Following initial automatic orienting to threat cues, directing attention away from threat (avoidance) would represent a strategic attempt to alleviate the anxiety elicited by threat-related stimuli (Mogg et al., 2004; Koster et al., 2006).

It has been proposed that the ability to reallocate attention in a flexible way after a stressful or traumatic event may be crucial in supporting a gradual decrease in intrusive re-experiencing and, consequently, a reduction of the attentional bias toward trauma-related cues (Verwoerd and Wessel, 2010). In this perspective, in the last decade an attention bias modification training (ABMT) has been developed, that aims at modifying biased attentional patterns and reduce distress symptoms (e.g., Bar-Haim, 2010; Hakamata et al., 2010; MacLeod and Mathews, 2012). ABMT is a computer-based protocol that implicitly trains individuals to attend away from threat stimuli. Most ABMT protocols have been adapted from well-established paradigms in experimental cognitive psychology, most notably the dot-probe task (MacLeod et al., 1986). In this task, each trial involves the simultaneous presentation of two stimuli (faces, pictures, or words), one threatening and one neutral (or positive), on a computer screen. After the offset of these stimuli, a probe (e.g., one or two dots, or a letter) appears in the spatial location of one of the two stimuli. The participant is required to respond to the probe, e.g., by pressing a key on the computer keyboard, as quickly as possible. In the classic version of this task, designed to measure attentional biases, probes appear with equal probability at the location of threat (congruent trials) and neutral stimuli (incongruent trials). Faster responses to a probe that replaces a threatening rather than a neutral stimulus are interpreted as preferential attention toward threatening information. The attentional bias can be reduced using this paradigm by systematically varying the proportion of trials in which the probe replaces the threatening and the neutral stimulus, that is, by having the probe replace the neutral stimulus on 100% of trials. This way, participants implicitly learn the association between the neutral stimulus and the target response and begin attending to neutral stimuli and away from threat stimuli. The effectiveness of the ABMT can be evaluated by removing the contingency between the stimulus and the probe, and examining whether participants who have undergone the protocol continue to demonstrate an attentional bias toward threat stimuli (see Bar-Haim, 2010).

The ABMT has been shown to be effective in reducing anxiety symptoms in individuals with high trait anxiety (see Lazarov et al., 2017, for effects related to implicit and explicit instructions), in patients with generalized social anxiety disorder (Schmidt et al., 2009), with obsessive-compulsive disorder (Najmi and Amir, 2010), and with generalized anxiety disorder (Hakamata et al., 2010). The effects of the training appear to be maintained even at follow up (see Schmidt et al., 2009). Of note, even a single session of ABMT has been reported to significantly reduce anxiety symptoms in anxious individuals (Amir et al., 2008), and film-related intrusive memories in healthy individuals who had watched a traumatic film (Verwoerd et al., 2012). Regarding individuals who experienced traumatic events, a randomized controlled study on combat veterans with chronic post-traumatic stress disorder (PTSD) showed that ABMT led to a reduction of post-traumatic symptoms with a moderately high effect size, and five out of six patients reported clinically significant improvement 1 week after the training (Schoorl et al., 2013). Nevertheless, it has to be noted that such reduction in symptomatology was not significantly larger compared to the control treatment. Kuckertz et al. (2014) evaluated the effectiveness of the ABMT as an adjunct training in patients with PTSD and found that patients reported significantly fewer post-traumatic and depressive symptoms post-treatment. More recently, Wald et al. (2017) showed that a single session of ABMT delivered before combat moderated the relationship between stress exposure and stress-related symptoms in a large group of soldiers (Wald et al., 2017).

However, in the past few years the effectiveness of the ABMT has been questioned in some reviews and meta-analyses (e.g., Emmelkamp, 2012; Mogoaşe et al., 2014; Cristea et al., 2015; Heeren et al., 2015). Inconsistent results may be due to methodological issues such as the use of different tasks (e.g., dot-probe or visual search), the use of different stimuli (e.g., words or pictures), the different number of training sessions (ranging from 1 to 28), the different symptom outcomes considered, and different instruments used to measure them (see Mogoaşe et al., 2014; Cristea et al., 2015). Importantly, a crucial issue is that most studies investigating the effectiveness of the ABMT have included individuals with no preexisting attentional bias toward trauma- or threat-related stimuli, or have not included a pre-training assessment of the attentional bias, thus failing to meet a fundamental assumption of the ABMT (Mogg et al., 2017). Overall, current evidence suggests that more research needs to be done to establish the potential of the ABMT as an effective standalone treatment or part of existing treatments (see Clarke et al., 2014).

The aims of the present study were twofold: first, to assess the presence of an attentional bias in workers who experienced a traumatic work-related accident; second, to evaluate the effectiveness of a single session of ABMT in modulating the attentional bias in injured workers.

## Materials and Methods

### Participants

Nineteen members of the Associazione Nazionale Mutilati e Invalidi del Lavoro (ANMIL) (17 males and 2 females) who had experienced work-related accidents were recruited. Inclusion criteria were the following: age-range between 18 and 50 years, time lapsed from the accident between 6 months and 5 years, degree of physical impairment between 19 and 70% [corresponding to a medium level of physical impairment, as assessed by the Istituto Nazionale per l’Assicurazione contro gli Infortuni sul Lavoro (INAIL), Italian Workers’ Compensation Authority]. Mean age, mean time lapsed from the accident, and mean degree of physical impairment are reported in **Table 1**.

**Table 1:** Socio-demographic characteristics, questionnaire scores, cognitive tests and Attentional Bias Score comparison between Injured workers and Controls.

Participants characteristics	Injured workers (*N* = 19)	Controls (*N* = 11)	U/χ^2^	*p*
Age (year)	43.00 (6.66)	42.45 (7.99)	102.00	*0.91*
Males (N, %)	17 (89)	8 (73)	1.41	*0.33*
Educational level (N, %)			3.44	*0.23*
Low	1 (5)	1 (9)		
Moderate	10 (53)	2 (18)		
High	8 (42)	8 (73)		
Mean time lapsed from the accident (years)	3.68 (1.60)			
Mean degree of physical impairment (%)	28.74 (8.23)			
STAI-Y1	35.37 (13.93)	33.09 (6.11)	104.50	*0.999*
STAI-Y2	38.74 (13.35)	37.36 (4.90)	91.50	*0.576*
BDI-II	9.74 (12.20)	5.91 (5.15)	98.00	*0.780*
PSS	11.32 (11.54)	6.18 (10.56)	58.50	*0.047*
WDQ	46.79 (16.29)	30.44 (14.75)	52.50	*0.025*
TMT-A (sec)	36.63 (17.90)	26.27 (7.63)	57.50	*0.043*
TMT-A (errors)	0.09 (0.30)	0.09 (0.30)	86.50	*0.438*
TMT-B (sec)	116.32 (53.02)	64.55 (17.82)	20.00	*0.0003*
TMT-B (errors)	0.26 (0.45)	0.95 (0.85)	45.00	*0.010*
d2 indexes				
Correct response (%)	89.21 (6.95)	90.30 (9.40)	87.00	*0.451*
Concentration performance	375.79 (97.00)	506.73 (78.29)	29.00	*0.001*
Fluctuation rate (in speed of processing)	13.37 (5.75)	12.09 (6.49)	74.50	*0.197*
Error distribution	0.03 (1.71)	0.68 (1.58)	71.50	*0.155*
Attentional Bias Score	10.78 (17.73)	-9.39 (14.95)	43.00	*0.008*


*Data are M (SD) of continuous and N (%) of categorical variables. Statistics are Mann–Whitney U for continuous and χ^2^ for categorical variables. STAI Y1/Y2, State-Trait Anxiety Inventory; BDI-II, Beck Depression Inventory, second edition; PSS, PTSD Symptom Scale; WDQ, Worry Domains Questionnaire; TMT A/B, Trail Making Test A/B form.*

The gender ratio in our sample of injured workers (89% male) fairly matched the gender distribution of work-related injuries in the European and Italian populations (78.5 and 79.6%, respectively; Eurostat, 2017).

A brief semi-structured interview was conducted to collect socio-demographic data (age and education) and a description of the type of accident. The type of accident could be classified as follows (number of participants in parenthesis): driving a car while on the job (6), being hit (6), being caught in, under or between something (2), fall at the same level or from an elevation (2), cut (2), lifting weights (1).

Eleven healthy individuals (eight males, three females) were recruited as control group. Inclusion criteria were the same as for the injured worker’s group, except for the absence of work-related accidents.

For both groups, exclusion criteria were: the presence of physical and psychological disorders unrelated to the accident; substance abuse; use of drugs or medications that could affect the individual’s ability to perform cognitive tasks; incapacity to give informed consent, traumatic brain injury, sensory (visual or hearing) loss. Specific questions of the semi-structured interview were aimed at collecting information relevant to the above exclusion criteria.

Injured workers and Controls did not differ for age, gender, and educational level (see **Table 1**).

After the administration of the semi-structured interview and the questionnaires, the cognitive tests and the pre-training dot-probe, injured workers (*N* = 19) were randomly assigned to a single session of ABMT (*N* = 10) or Attention Control Condition (ACC) (*N* = 9). Injured workers undergoing ABMT showed no difference from injured workers undergoing ACC training in terms of sociodemographic (age, gender, educational level), psychological (STAI-Y1, STAI-Y2, BDI-II, PSS, WDQ), cognitive (TMT-A, TMT-B, d2) variables and Attentional Bias Score (all *p*’s > 0.08).

The study was conducted in accordance with the Declaration of Helsinki and approved by the local Ethics committee (Protocol No. 2021).

### Questionnaires

The State-Trait Anxiety Inventory (Spielberger, 1983; Italian version by Pedrabissi and Santinello, 1989) was used to assess self-reported state (Y1) and trait (Y2) anxiety symptoms. Raw scores on each scale range between 20 and 80, with higher scores reflecting higher levels of anxiety.

The Beck Depression Inventory-II (BDI-II; Beck et al., 1996; Italian version by Ghisi et al., 2006) was used to evaluate the severity of depressive symptoms in the past 2 weeks. Higher scores (range: 0–63) correspond to more severe depressive symptoms.

The PTSD Symptom Scale (PSS; Foa et al., 1993) was used to assess the severity of post-traumatic symptomatology on three subscales, i.e., re-experiencing, avoidance, and arousal. The total score reflects the severity of PTSD symptoms.

Worry was assessed by the Worry Domains Questionnaire (WDQ Tallis et al., 1992), a self-report questionnaire suitable for use on non-clinical adult populations, evaluating worry contents on five subscales (relationship, lack of confidence, aimless future, work incompetence, and financial). The total score gives an indication of worry frequency, and the subscales provide information with respect to worry content.

### Cognitive Tests

The following instruments were used:

Trail making test (TMT; Reitan, 1958; Italian version by Giovagnoli et al., 1996): it assesses visual search abilities and executive functions, and provides measures of perceptual, motor and set-shifting skills. It is made up of two parts: A (numbers) and B (numbers and letters). TMT-A requires an individual to draw lines sequentially and connect 25 encircled numbers distributed on a sheet of paper. In TMT-B the individual must alternate between numbers and letters (e.g., 1, A, 2, B, 3, C, etc.). The individual’s performance is evaluated as total time (in seconds) required to complete the task, and error rates.

d2 test (Brickenkamp and Zillmer, 1998): it provides a measure of attention span and concentration. It is composed of 14 successive timed trials, each including 47 items that can be targets (the letter “d” with two strokes) or visually similar stimuli (e.g., the letter “d” with one, three or four strokes, the letter “p” with one, two, three, or four strokes). During each trial, participants are required to cancel out as many targets as they can in 20 s, as fast and with as few errors as possible. Different scores can be computed, reflecting distinctive features of performance, namely the total correctly processed stimuli (total stimuli processed minus total errors made), concentration performance (the difference between the number of correctly canceled items minus the number of incorrectly canceled items), fluctuation rate (calculated as the maximum total stimuli processed in a single trial minus minimum total number of items processed in a single trial), and error distribution (average errors for the last four trials minus average errors for the first four trials).

### Dot-Probe Task

A modified dot-probe task was used to measure the attentional bias both before and after the training. Thirty digitized picture stimuli depicting neutral stimuli (household objects) and 30 trauma-related pictures collected and validated from our group in previous studies (see Buodo et al., 2011), depicting victims of accidents in occupational contexts (e.g., a worker falling from scaffolding in a construction setting; a worker being caught under industrial machinery) were used. Pictures were divided in three sets including 10 neutral and 10 trauma-related pictures each and used separately during the three phases of the experiment (pre-training dot-probe, training, and post-training dot-probe). The pictures were adjusted to fit the dimension of 300 × 300 pixels and were presented 50 pixels apart (above or below) from the center of the screen.

Each trial (*N* = 200) started with the presentation of a fixation point in the center of a gray background for 500 ms. Then, a pair of picture stimuli, one trauma-related and one neutral, or both neutral, were presented above and below the center of the screen for 500 ms, followed by a target probe that appeared at the location of one of the two pictures. Target stimuli consisted of a “+” or a “×” symbol and remained on the screen until a response was given. Participants were instructed to discriminate the two arithmetic operators by a vocal response (the Italian words for the addition and the multiplication signs have similar pronunciations and length; see Buodo et al., 2011, for a similar procedure in an emotional interference task). The inter-trial interval randomly varied between 500 and 1000 ms. Targets were presented equally often above or below the fixation point and were equally often a “+” or a “×.”

When the picture pair consisted of one trauma-related and one neutral picture, Congruent trials (*N* = 80) were those where the target was presented at the location of the trauma-related picture. Instead, Incongruent trials (*N* = 80) were those where the target appeared at the location of the neutral picture. On Neutral trials (*N* = 40) both pictures were neutral, and the target could follow on either location. Picture category (trauma-related or neutral), location (above or below the center of the screen) and target type (“+” or “×”) were counterbalanced; each trial was repeated twice.

The task was administered using a Notebook HP Pavilion dv2000, Intel Core 2 Duo (1.66 GHz) processor, with a 14.1′′ monitor and 1280 × 800 resolution, running “E-prime 1.1” software (Psychology Software Tools, Inc., Pittsburgh, PA, United States) for picture presentation.

Vocal reaction times (RTs) were recorded using a microphone, connected to a Response Box (Psychology Software Tools, Inc., Pittsburgh, PA, United States). Not naming the correct target, and unrecognizable or missing responses were computed as errors.

### Training Tasks

The ABMT was developed to train the participants’ attention toward neutral pictures using a modified dot-probe procedure. The training consisted of 400 trials divided into four blocks. The procedure was identical to that of the pre-training dot-probe task, except that pictures were taken from a different set than that used in the pre- and post-training dot-probe task. Also, the target always appeared at the location of the neutral picture. Specifically, 320 trials were Incongruent and 80 were Neutral. Picture category (trauma-related or neutral), location (above or below the center of the screen) and target type (“+” or “×”) were counterbalanced; each trial was repeated four times.

The ACC consisted in a dot-probe task where the target could appear with equal probability at the location of the trauma-related or the neutral picture (160 Congruent trials, 160 Incongruent trials, and 80 Neutral trials). Picture category (trauma-related or neutral), location (above or below the center of the screen) and target type (“+” or “×”) were counterbalanced; each trial was repeated twice.

### Procedure

Before participation in the study, all subjects read and signed an informed consent form. After the semi-structured interview and the questionnaires, the cognitive tests (TMT-A, TMT-B, and d2) were administered, and the pre-training dot-probe task was run (duration about 10 min). Then, only injured workers (*N* = 19) were randomly assigned to receive either the ABMT (*N* = 10) or the ACC (*N* = 9) (duration about 20 min), followed by the post-training dot-probe task (duration about 10 min). At the end of the session, participants were thanked and debriefed. Specifically, all injured workers were provided with information on pre- and post-training assessments and about the ABMT and the ACC. Injured workers in the ACC group were given the possibility to undergo an ABMT protocol.

#### Data Analysis

In the dot-probe tasks, all trials with errors (i.e., not naming the correct target) and unrecognizable or missing responses, were removed (average 0.67%). Following the procedure reported by Van Bockstaele et al. (2012), outlier responses were removed first as RTs faster than 150 ms and slower than 1500 ms (12.6%), and then as individual RTs deviating more than three SDs from the individuals’ mean (2.1%).

Attentional Bias (AB) scores were calculated for both the pre-training and the post-training task as mean RTs on Incongruent trials minus mean RTs on Congruent trials. Positive AB scores indicate vigilance toward trauma-related stimuli (faster RTs when the probe appeared on the location of trauma-related stimuli), whereas negative AB scores indicate attentional avoidance (slower RTs when the probe appeared on the location of trauma-related stimuli).

Mann–Whitney *U*-tests and χ^2^s for independent groups were performed to compare Injured workers to healthy Controls in terms of age, gender, educational level, scores on questionnaires (STAI-Y1, STAI-Y2, BDI-II, PSS, WDQ), performance measures on the cognitive tasks (TMT-A, TMT-B, d2) and AB score.

Moreover, Pearson’s correlation coefficients were calculated to test whether pre-training AB scores could be related to age and to questionnaire scores (STAI-Y1, STAI-Y2, BDI-II, PSS, WDQ) in the Injured workers group. Univariate analysis of variances (ANOVAs) were also used to evaluate AB scores as a function of gender (males vs. females) and educational level (low, moderate, high).

As a second step, mixed ANOVA was conducted to compare AB scores in injured workers who underwent the ABMT and the ACC. Specifically, an ANOVA with Group (ABMT vs. ACC) as a between-subject factor, and Time (pre- vs. post-training) as within-subject variable was performed. Partial eta-squared ($\eta_{p}^{2}$) was reported as a measure of the effect size. Significant main effects and interactions (*p* < 0.05) were followed by Fisher *post hoc* comparisons to identify specific differences.

Data were analyzed using STATISTICA software version 6.1 (StatSoft, Inc., Tulsa, OK, United States).

## Results

The analyses performed on questionnaire scores showed no significant differences between Injured workers and Controls in self-reported state and trait anxiety and depressive symptoms. Injured workers scored significantly higher than Controls in self-reported symptoms of PTSD and worry (see **Table 1**).

Injured workers performed significantly worse than Controls in the TMT-A [sec], TMT-B [sec, errors], and d2 [concentration performance]. No significant differences emerged in the other performance parameters (all *p*’s > 0.15; see **Table 1**).

**FIGURE 1:**
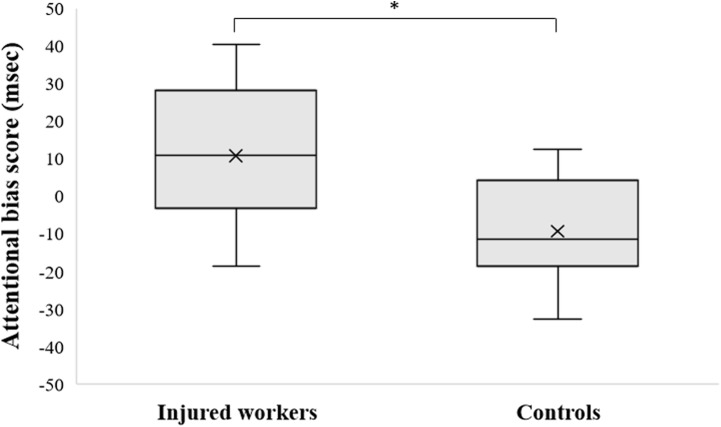
Attentional Bias score (AB) values (msec) in Injured Workers and Controls. Cross represents the mean value, central band represent the median, bottom and top of the box represent the first and third quartile respectively, whiskers reflect the minimum and maximum of all the data. ^∗^*p* < 0.01.

Injured workers showed significantly higher AB scores than Controls in the pre-training dot-probe task (see **Figure 1**).

AB scores were unrelated to age and questionnaires scores (STAI-Y1, STAI-Y2, BDI-II, PSS, WDQ) (all *p*’s > 0.08). No differences emerged between males and females (*F*_[1,17]_ = 0.03, *p* = 0.87, $\eta_{p}^{2}$ = 0.05) nor among different educational levels (*F*_[1,17]_ = 0.41, *p* = 0.67, $\eta_{p}^{2}$ = 0.11).

### Attentional Training Tasks

Analysis of variance on AB scores in the pre- and post-training dot-probe tasks yielded a significant main effect of Time (*F*_[1,17]_ = 6.74, *p* < 0.05, $\eta_{p}^{2}$ = 0.28), showing a reduction in AB scores from pre- to post-training.

**FIGURE 2:**
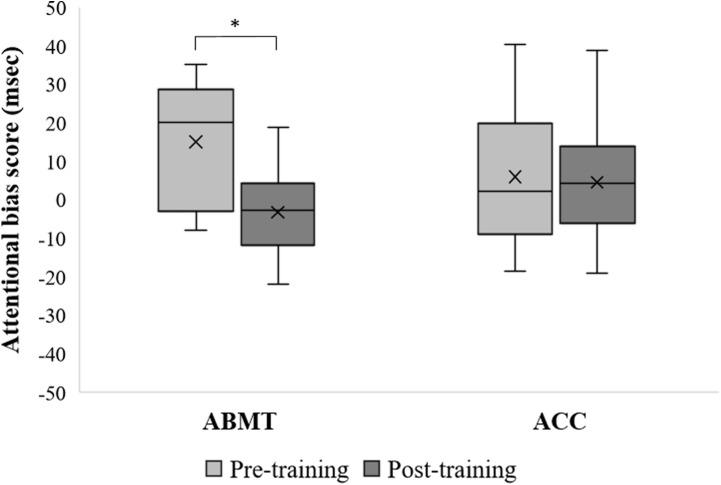
Attentional Bias score (AB) values (msec) in attention bias modification training (ABMT) and Attention Control Condition (ACC) group, pre- and post-training. ANOVA revealed a significant Group × Time interaction effect (*p* < 0.05). Cross represents the mean value, central band represent the median, bottom and top of the box represent the first and third quartile respectively, whiskers reflect the minimum and maximum of all the data. ^∗^*Post hoc* Fisher’s LSD comparisons, *p* < 0.01.

Importantly, a significant interaction between Group and Time emerged (*F*_[1,17]_ = 4.93, *p* < 0.05, $\eta_{p}^{2}$ = 0.22; see **Figure 2**). *Post hoc* analysis revealed that there was a significant reduction in AB scores from pre- to post-training in the ABMT group (*p* < 0.01), while no difference in AB scores from pre- to post-training emerged in the ACC group (*p* = 0.80). No significant effect of group emerged (*F*_[1,17]_ = 0.01, *p* = 0.93, $\eta_{p}^{2}$ = 0.0005).

## Discussion

The aims of the present study were twofold. The first was to evaluate whether individuals who underwent a work-related traumatic injury would show an attentional bias toward trauma-related pictures compared to workers who never experienced a traumatic injury. The second was to test the possibility to modify the attentional bias in injured workers through a single-session ABMT.

The obtained findings indicate that individuals who experienced a traumatic work-related accident show an attentional bias toward trauma-related stimuli. In other words, injured workers were faster when they had to respond to a target that appeared in the spatial position of a trauma-related picture, compared to when they had to respond to a target that appeared in the position of a neutral picture. It has to be noted that AB scores were unrelated to post-traumatic symptoms (as measured by the PTSD Symptom Scale), as well as to age and questionnaires scores. In our group of injured workers no one showed clinical symptoms of PTSD, and this could be explained by the fact that the questionnaire could not evidence subtle cognitive-emotional changes such as those that emerged in the dot-probe task. These results fit with previous findings showing a lack of correlation between PTSD symptoms severity and RTs in an emotional interference task (Buodo et al., 2011).

Our findings are in line with previous studies that have found an attentional bias characterized by faster RTs to trauma-related pictures in individuals who underwent a traumatic event (e.g., Williams et al., 1996; Bar-Haim et al., 2007). According to the cognitive models of PTSD (e.g., Foa et al., 1989; Litz and Keane, 1989), patients with PTSD are hypervigilant toward threat cues because trauma-related materials are easily activated in fear networks which are encoded in long-term memory at the time of trauma. As a result, these individuals are faster to detect and process threat-relevant stimuli, they are more easily distracted from other tasks by these stimuli, and they display enhanced allocation of processing resources to such information (Foa et al., 2006). Enhanced attention to trauma-relevant stimuli leaves fewer attentional resources available for the processing of emotionally neutral information. Since the range of potential trauma reminders is acknowledged to be quite broad for traumatized individuals, an attentional bias toward trauma-related cues is expected to have deleterious effects even for individuals who are able to get a new job in a different setting than that of the accident.

Injured workers scored higher on questionnaires measuring post-traumatic symptoms and worry compared to controls, while no differences between groups emerged for anxiety and depressive symptoms. These results, although consistent with those of previous studies in which injured workers were found to report the presence of PTSD symptoms, fail to replicate the evidence that individuals who underwent work-related accidents also report more anxiety and depressive symptoms than controls (Novara et al., 2009; Ghisi et al., 2013). As a possible explanation of this discrepancy, it should be noted that in previous studies the PTSD symptomatology, i.e., reexperiencing, avoidance, detachment, excessive arousal and hypervigilance, reported by participants was overall more severe than that endorsed by participants in our study. In line with previous results (Buodo et al., 2011), injured workers showed worse performance both in visuospatial search (i.e., TMT-A), switching ability (i.e., TMT-B) and sustained attention (i.e., d2) as compared with controls. Thus, it is noteworthy that even in the absence of significant co-occurring anxiety and depressive symptoms, victims of workplace accidents can indeed develop emotional and cognitive dysfunctions, as indicated by PTSD symptoms and impaired cognitive performance.

A single-session training aimed at directing attention away from trauma-related stimuli was effective in reducing the attentional bias in our sample of injured workers. A substantial reduction of attentional bias scores was observed from pre- to post-training, as measured with the dot-probe task, only in injured workers who underwent the ABMT, while no differences emerged in injured workers who underwent the ACC. These results are in line with previous studies where the ABMT was found to be effective in reducing/modifying the attentional bias in healthy (Van Bockstaele et al., 2012) and subclinical obsessive-compulsive and socially anxious individuals (Amir et al., 2008; Najmi and Amir, 2010). To date, this is the first study to our knowledge focusing on the modification of the attentional bias in individuals who underwent traumatic accidents in the workplace. Although the mechanism by which the ABMT may induce a change in the attentional bias is not entirely clear (see Browning et al., 2010; Heeren et al., 2013; Mogg et al., 2017), it can be hypothesized that the ABMT was effective in promoting attentional disengagement of attention from trauma-related stimuli.

The findings of the presence of an attentional bias in injured workers and of the effectiveness of a single session of ABMT in reducing such bias is of great importance, given that the presence of an attentional bias has been found to be associated with worse performance in cognitive tasks. It is particularly relevant to note that an occupational accident in the previous 3 months has been associated with a higher risk of experiencing a new accident (Probst and Brubaker, 2001). Reduced attention and/or increased intrusive memories and thoughts related to the traumatic event might be causal factors involved in the increased probability of further accidents. As shown in this and in previous studies, individuals who underwent traumatic work-related accidents show higher interference from traumatic cues, tend to direct their attention toward trauma-related contents, and have difficulty disengaging attention from these contents (e.g., Buodo et al., 2011). Enhanced attention toward trauma-related cues may play a role in the maintenance of high levels of worry and stress-related symptoms (Wald et al., 2011). More importantly, the attentional bias may play a central role in the maintenance and exacerbation of cognitive deficits, which in turn could lead to difficulties in returning to previous occupational roles or increase the risk of recurrence of job accidents.

The obtained findings should be considered as preliminary in light of the fact that the sample was relatively small, thus limiting statistical power. However, and importantly, our study provided evidence that a single session of ABMT was effective in reducing the attentional bias in injured workers who do show an attentional bias toward trauma-related stimuli. In order to increase the potential use of the procedure in work and clinical settings, multiple follow-ups would be crucial to evaluate long term cognitive-affective changes after a single session ABMT. In addition, future studies should address the issue of the generalizability of the effect of the training on other cognitive functions. Indeed, previous studies found mixed results, with some reporting improvement in tasks which are similar to the one used for the training (see Amir et al., 2008, 2009; Verwoerd et al., 2012), and others finding no generalization after a single session ABMT (see Van Bockstaele et al., 2012). Future studies are warranted to test whether a multiple session ABMT could have clinical utility by reducing cognitive (e.g., executive functions, memory, sustained attention) and distress symptoms (e.g., post-traumatic symptoms) in individuals who underwent a work-related accident.

## Conclusion

The preliminary findings of the present study support the presence of an attentional bias toward trauma-related stimuli in injured workers and suggest the possibility of reducing this bias through a short behavioral intervention.

## Author Contributions

GB, EP, and DP contributed conception and design of the study. EP gathered the data and organized the dataset. GB, EP, and SMB performed the statistical analysis. All authors contributed to manuscript revision, read and approved the submitted version.

## Conflict of Interest Statement

The authors declare that the research was conducted in the absence of any commercial or financial relationships that could be construed as a potential conflict of interest.
